# Identifying the components of clinical vignettes describing Alzheimer’s disease or other dementias: a scoping review

**DOI:** 10.1186/s12911-015-0179-x

**Published:** 2015-07-15

**Authors:** Harkanwal Randhawa, Aalim Jiwa, Mark Oremus

**Affiliations:** Faculty of Health Sciences, McMaster University, 1280 Main Street West, Hamilton, ON Canada; School of Public Health and Health Systems, University of Waterloo, 200 University Avenue West, Waterloo, ON Canada

**Keywords:** Vignette, Alzheimer’s disease, Dementia

## Abstract

**Background:**

Clinical vignettes are often used to elicit information about health conditions in research studies. This review summarizes the components of clinical vignettes describing Alzheimer’s disease (AD) or other dementias. The purpose is to provide recommendations for the development of standardized vignettes that may be used in future studies.

**Methods:**

MEDLINE, EMBASE, PsycINFO, ASSIA, CINAHL were searched from their inception to June 2014. Primary English-language studies employing vignettes to describe AD or similar disorders (including other dementias and Parkinson’s disease) were included in the review. Included studies had to describe the content of the vignettes in the published manuscripts. The characteristics of the included studies and the vignettes were extracted in tabular form and summarized qualitatively.

**Results:**

Forty-two studies were included in the review. Twenty-four of the studies contained at least one AD vignette, 11 had vignettes focusing on non-AD dementias, and seven contained vignettes describing conditions other than dementia. In total, 58 vignettes were obtained from the 42 included studies.

**Conclusions:**

Key aspects to consider when constructing vignettes for AD or other dementias include writing the vignettes from a third-person perspective and presenting hypothetical patients as being at least 65 years of age. Researchers should develop standardized vignettes for use across studies.

**Electronic supplementary material:**

The online version of this article (doi:10.1186/s12911-015-0179-x) contains supplementary material, which is available to authorized users.

## Background

Dementia is a condition affecting higher cortical and cognitive functions, including memory, learning capability, judgment, emotional control, and social behaviour. Alzheimer’s disease (AD) is the most common form of dementia in elderly adults, accounting for about sixty percent of cases of dementia [1, 2]. AD is an irreversible and progressive form of brain disease that eventually leads to an inability to carry out simple activities of daily living [3]. A gradual onset of memory impairment, followed by deterioration in other cognitive areas (e.g., language, abstraction, construction), is characteristic of AD.

In research studies, the signs and symptoms of diseases such as AD can be written as brief descriptions that illustrate how the diseases manifest themselves in patients. The descriptions, known as clinical vignettes, may be used as tools to measure a diversity of variables, including quality-of-life (QoL), public perceptions of disease, and variations in healthcare practice across jurisdictions [4, 5]. For example, Osborne et al. used a vignette that described an ‘average’ person with schizophrenia to generate health utility index scores for a QoL study [6]. In another project, Hudelson employed clinical vignettes to analyze medical students’ and physicians’ capacities to identify sociocultural factors that affect health and health care in persons with conditions such as hypertension or tuberculosis [7]. Alexander and Becker formally define vignettes as “short descriptions of a person or a social situation which contain precise references to what are thought to be the most important factors in the decision-making or judgment-making process of respondents (p. 94)” [8].

In AD, vignettes are especially important for obtaining proxy information in place of persons with the disease, whose levels of cognitive deterioration may prevent them from providing valid responses to certain types of data collection instruments. For example, persons who are beyond the mildest state of AD may be cognitively incapable of assessing their QoL using an instrument such as the EQ-5D [9]; the task of estimating QoL becomes more difficult as cognitive function worsens [10]. Also, caregivers’ proxy estimates of their care recipients’ QoL tend to be lower than care recipients’ own estimates [11, 12]. Members of the general population could become an alternative source of proxy QoL estimates for persons with AD [13]. To elicit these proxy estimates, members of the general population could read a vignette describing AD and they could answer the questions on the EQ-5D as if they had AD based on the vignette they just read.

An important consideration to make when designing a vignette-based study is the content of the vignettes. Vignettes should present realistic situations to maximize study validity [14, 15]. However, considerations such as length, wording, and target audiences mean that the scenarios described in the vignettes are often only partial representations of the challenges and symptoms that accompany medical conditions [4]. Studies in the same disease or treatment domain might lack comparability if they are based on different vignettes.

Well-designed vignettes can generate results that permit researchers to make valid inferences about the variables under study [16] In AD or dementia, filming actors or real-life cases (e.g., persons with AD or dementia) is an alternative means of addressing some of the research questions that one might otherwise use vignettes to study. However, such films would still likely be partial representations of the totality of the medical condition, and the ethical and resource implications of creating the films might eclipse any incremental benefits related to the validity of the collected data.

We conducted a scoping review to identify studies that employed vignettes to describe AD or other dementias. We summarized vignette characteristics, purposes, and foci. No previous reviews have been undertaken on this topic. Our review provides recommendations on how to construct clinical vignettes for AD or other dementias in future research projects.

## Methods

### Data sources and search strategy

We used MEDLINE, EMBASE, PsycINFO, Applied Social Sciences and Abstracts (ASSIA), and Cumulative Index to Nursing and Allied Health Literature (CINAHL) to search the literature from each database’s inception to June 2014. The search strategy combined “vignette”, “Alzheimer’s disease”, “Dementia”, and a list of similar disorders. We incorporated similar disorders into the review to obtain a broader sample of vignettes from which to draw recommendations about the construction of vignettes for AD or other dementias. We felt the design and content of as many vignettes as possible should be studied to optimize our recommendations. The complete search strategy, developed with the assistance of a medical librarian, is shown in Additional file 1 along with a comprehensive list of similar disorders.

Five sources were searched to develop a comprehensive list of the common outcomes, symptoms, and behaviours of persons with AD or other dementias [1–3, 17, 18]. After creating the list, the five sources and the MedlinePlus Medical Encyclopedia were searched for an extensive list of similar disorders that had at least three outcomes, symptoms, or behaviours that resembled AD or other dementias [19]. At least one of the similar characteristics had to be a decline in cognitive function relating to memory, language, recognition, motor function, or executive function (i.e., planning, organizing, abstracting). Our approach led to three groups of vignettes in the review: vignettes for AD, vignettes for other dementias, and vignettes for disorders that are similar to AD.

### Study selection criteria

The articles identified through the database searches were included in the review if they satisfied all of the following criteria:

1. Reported data from a primary study;
2. Used vignettes describing AD, another form of dementia, or a similar disorder in their study methods;
3. Contained the text of the vignettes used in the research, or provided descriptions of the content of the vignettes; and
4. Published in English.

We excluded case reports, commentaries, editorials, letters, and reviews from our study.

Recent work from the Canadian Agency for Drugs and Technologies in Health does not show that limiting literature searches to English-language articles will bias the results of systematic reviews [20].

### Study screening and data extraction

Two reviewers (HR and AJ) independently screened each citation found in the literature search. After removal of duplicate citations, the reviewers applied the inclusion and exclusion criteria by reading the title and abstract of each article. Articles that met the inclusion criteria, or which could not be fully assessed based on the information available in the title and abstract, were promoted to full-text screening. At full-text screening, the reviewers read the entire article to assess the inclusion and exclusion criteria. Disagreements between reviewers were resolved by consensus. We extracted the following information from the included studies: vignette development, study objectives, vignette administration methods, point of view used to describe patients in vignettes (i.e., first-, second-, third-person), outcomes/symptoms described in the vignettes, and the characteristics of the hypothetical patients featured in the vignettes.

## Results

### Selection of studies

**Fig. 1:**
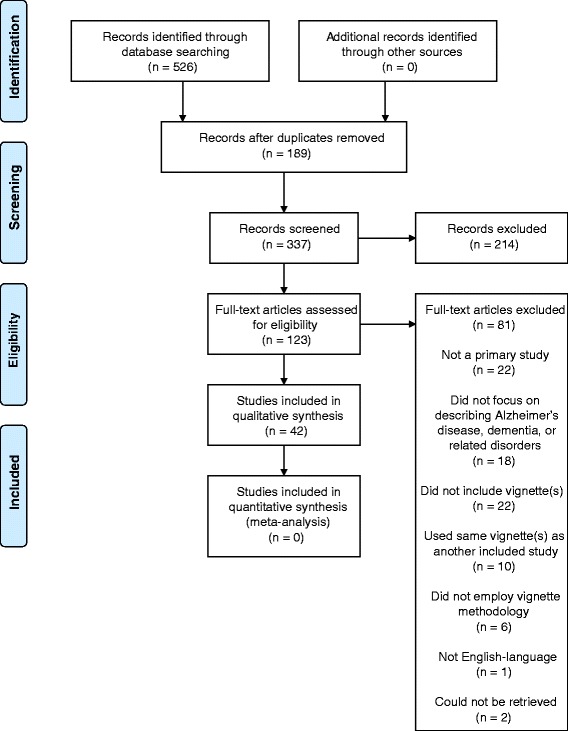
PRISMA flowchart for the selection of articles

Five hundred and twenty-six citations were identified through the literature search. After removing 189 duplicates, we screened the titles and abstracts of 337 citations and removed 214 citations. Of the 123 citations promoted to full text screening, 42 met the inclusion criteria and were retained in the review (Fig. 1) [13, 21–61]. Five of the removed citations [62–66] contained vignettes that were virtually identical to the vignettes that were reported in two included studies [57, 59].

Forty studies featured at least one published vignette; two additional studies [24, 40] described the content of vignettes without reproducing the actual text of these vignettes. Twelve studies contained more than one vignette [13, 25, 26, 28, 33, 36, 37, 45–47, 50, 57]; in total, 58 vignettes were identified among the 42 studies.

### Study characteristics

Studies were summarized into three tables (Additional file 2: Table S1, S2, and S3) based on the disease described in the vignettes. Additional file 2: Table S1 contains all 24 studies that included at least one vignette on AD [13, 21–23, 25, 29–31, 33, 36, 39, 41–43, 46–52, 56–59], Additional file 2: Table S2 contains 11 studies whose vignettes only focused on other dementias [24, 26, 28, 37, 40, 45, 49, 53, 54, 60, 61], and Additional file 2: Table S3 contains 7 studies whose vignettes focused on conditions other than AD or dementia [27, 32, 34, 35, 38, 44, 55].

#### Development of vignettes

Of the studies included in the review, only 12 (29 %) described the development of the vignettes [21, 22, 34, 35, 37, 42, 50, 55–59]. In general, the authors of these 12 studies tested face validity, content validity, and feasibility by asking experts in the field (e.g., clinicians with geriatric and/or psychiatric expertise) to review the vignettes. The authors of four of the 12 studies pilot-tested the vignettes on researchers [21], healthcare providers (e.g., nurses) with geriatric and psychiatric expertise [34, 35], or experts in dementia (professional background not identified in the published article) [42]. No authors reported testing vignettes on persons with AD or their caregivers.

#### Study objectives

The primary objectives of the included studies varied widely. However, most aimed to evaluate patients’ attitudes and beliefs regarding the health conditions that were described in the vignettes. For example, Kalaitzaki et al. used vignettes to assess emotional reactions to AD in health professionals, laypersons, and graduate students [42]. Additionally, two studies used vignettes to obtain proxy QoL estimates from community-dwelling adults in place of persons with AD [13, 55].

The principal use of the vignettes in the included studies most closely maps onto what Aguinis and Bradley call the ‘paper people studies’ type of experimental vignette methodology [67]. For this methodology, researchers provide participants with vignettes in written form and ask the participants to make a decision, judgment, or choice that flows from the content of the vignettes.

#### Administration of vignettes

The majority of the studies administered the vignettes to a sample of participants from the general public or to healthcare professionals (frequently physicians and nurses).

The means of administering the vignettes to participants were described in 29 [13, 21, 22, 24, 28–33, 38, 43–50, 52–61] of the 42 studies: 12 (41.4 %) [13, 21, 22, 28, 30, 31, 33, 43, 47, 56, 57, 59] presented vignettes to participants in face-to-face interviews, five (17.2 %) [44, 46, 50, 58, 60] presented vignettes over the telephone, nine (31 %) [24, 29, 32, 38, 49, 52, 53, 55, 61] presented vignettes through the mail, and three (10.3 %) [45, 48, 54] presented vignettes through small group discussions. The remaining thirteen studies did not specifically explain how the vignettes were administered to participants; however, some of these studies did mention a setting within which the administration of the vignettes took place (e.g., during staff meetings [27] or physician grand rounds [51]).

The vignettes were used to evaluate a diversity of outcomes, including social distance (i.e., the perceived degree of separation between various societal groups such as ethnic groups or social classes) [23], treatment options [29], emotional reactions [42], and QoL [13]. These outcomes were measured using validated instruments (e.g., EQ-5D-5 L [9, 13], Quality-of-life – Alzheimer’s Disease scale [13, 68], Perceptions of Restraint Use Questionnaire [43, 69]) or ad hoc questionnaires developed by the authors (e.g., a five-point scale measuring factors determining physicians’ decision making processes [51], a five-point Likert-type scale measuring information-seeking, information-giving, and involvement in patient-physician interactions [60]).

#### Vignette point of view

Six (10.3 %) of the 58 vignettes were presented to study participants in the second-person using phrases such as “If you have Alzheimer’s disease.” Fifty-two (89.7 %) were presented to participants in the third-person using names or pronouns (e.g., “Mr. X”, s/he) to label the hypothetical patients described in the vignettes [13, 32, 33].

#### Frequency of symptoms described in the vignettes

The 20 most frequently recurring symptoms presented in the vignettes are reported in Table 1. The five most common symptoms in the AD vignettes include deterioration of memory, changes in mood, difficulty with activities of daily living, signs of aphasia (i.e., an inability to understand or express speech), and signs of agnosia (i.e., a loss of ability to recognize persons, objects, sounds, and smells). The six most commonly recurring symptoms for non-AD dementias are deterioration of memory, changes in mood, disorientation, signs of aphasia, difficulties with activities of daily living, and withdrawal from social activities (the latter three symptoms shared the same frequency, so we reported six rather than five symptoms). The five most common symptoms for the non-AD/non-dementia disorders are deterioration of long-term memory, deterioration of short-term memory, signs of aphasia, difficulty with activities of daily living, and disorientation.

**Table 1:** Frequencies of common symptoms and behaviours described in vignettes

	AD Vignettes						
Symptom/Behaviour	Mild	Moderate	Severe	All AD^a^	Non-AD Dementia	Other Conditions	All Vignettes
	(*n* = 4)	(*n* = 4)	(*n* = 4)	(*n* = 33)	(*n* = 16)	(*n* = 9)	(*n* = 58)
Memory Deterioration/Forgetfulness	4	3	1	20	12	7	39
Short-term Memory Deterioration	2	1	1	7	4	5	16
Signs of Aphasia	1	2	1	8	5	4	17
Signs of Agnosia	0	1	1	8	1	2	11
Signs of Apraxia	0	0	0	2	2	1	5
Delusional Behaviour	0	1	1	4	1	0	5
Wandering Behaviour	0	0	1	3	2	1	6
Difficulty with Activities of Daily Living	1	3	1	10	5	4	19
Mood Changes (agitation, aggression, irritability, depressiveness, anxiety)	1	2	4	15	9	2	26
Declining ability to Concentrate	2	0	0	3	1	1	5
Disorientation (either time, place, or situation)	0	1	1	5	6	4	15
Denial of Condition	0	0	0	2	1	1	4
Incontinence	0	0	0	4	1	0	5
Declining Directional Ability	1	0	0	4	2	3	9
Repetition of Self	0	2	0	4	4	0	8
Withdrawal from Social Activities	2	1	0	3	5	0	8
Confusion	1	0	0	4	4	2	10
Inappropriate/Ill-mannered Behaviour	0	0	0	2	0	0	2
Difficulty Maintaining Self/Self-Appearance	0	2	1	3	3	1	7
Paranoia or Suspicion	0	0	1	2	3	2	7

Notes: Numbers represent the frequency of symptom/behaviour in vignette category, *AD* Alzheimer’s disease, aphasia represents as inability to understand or express speech, agnosia presents as a loss of ability to recognize persons, objects, sounds, and smells, and apraxia presents as a lack of ability to execute purposeful movements

^a^33 vignettes focused on AD, but only 12 explicitly stated the stage of AD described

### Characteristics of vignette patients

#### Vignette patient age

Fourteen vignettes did not present the ages of their hypothetical patients [21, 26, 29, 37, 44, 45, 48, 50, 54, 56]. Age was not applicable for six other vignettes because they were written in the second-person point-of-view [13, 32, 33]. In one study, the authors varied the age of patients substantially (i.e., 68 or 28 years of age) to determine whether older versus younger patients were more likely to be diagnosed with a cognitive disorder [27]. The mean (standard deviation [SD]) for patient age among the 33 vignettes describing AD or another dementia, and reporting age, was 74 (9) years; and the mean (SD) for patient age among the four vignettes focused on similar disorders, and reporting patient age, was 69 (14) years. We excluded the study by Ciliberto et al. from our computation of mean age [27].

#### Vignette patient sex

The sex of the hypothetical patients in the vignettes was not given for two [29, 44] vignettes and was not applicable for the six vignettes (published in three articles) written in the second-person [13, 32, 33]. Of the 50 remaining vignettes, nine (18 %) (published in seven articles) randomly assigned the sex of the hypothetical patient [22, 23, 28, 46, 47, 55, 56], 23 (46 %) described the sex as female, and 18 (36 %) described the sex as male. Stratified by vignette group, of the 41 hypothetical patients assigned a sex: 10 patients were female and 14 patients were male for AD; 10 patients were female and three patients were male for non-AD dementias; and three patients were female while one patient was male in vignettes for other conditions.

## Discussion

### Development and content of vignettes

Only a small proportion of the included studies (*n* = 12) reported on the development of the vignettes. Without this information, readers cannot assess whether the authors adequately validated the content of the vignettes. Since clinical vignettes are intended to accurately depict health conditions and evoke responses from study participants, the content validity of the vignettes is of paramount importance. Inaccurate vignettes will not effectively portray the characteristics of a disease and will lead to potentially biased data collection. For example, QoL estimates based on a vignette that portrays only the worst possible symptoms of disease will be biased downward. Future studies should describe the process of developing new vignettes. In the case of vignettes borrowed from other studies, the authors should report on how these vignettes were developed in the other studies.

In addition to explicit methods, another important consideration when developing vignettes is the degree to which the new vignettes should be similar to existing vignettes. A study might require the development of new vignettes because of the population under investigation (e.g., members of the general public should read vignettes written in lay language, whereas vignettes intended for nurses or clinicians might contain professional jargon). New vignettes may also be necessary to describe a novel treatment (e.g., a disease-modifying medication for AD). However, researchers should attempt to standardize the mix of symptoms and disease characteristics described within vignettes that pertain to the same health conditions [4]. Such standardization will enhance comparability between studies and encourage reproducibility of results.

### Patient age and sex

The mean age for patients, as described in the vignettes, was 74 years for the vignettes about AD and other dementias and 69 years for the vignettes about other disorders. These ages reflect the fact that both AD and dementia are rare in persons under the age of 60 [1, 70]. Estimates have found that the highest prevalence of AD is above the age of 75 years [71, 72], with prevalence increasing with age [73]. Patient age is an important component of clinical vignettes for AD because it is one of the strongest risk factors for the disease. Additionally, patient age helps to situate the context of the disease. For example, older patients may become more dependent on others to help with particular activities of daily living (ADL) over time. Vignettes should therefore describe hypothetical patients who are at least 65 years of age.

Patient sex is less important to capture in clinical vignettes for AD. Sex does not affect the presentation and progression of the disease. Researchers may wish to match the sex of the hypothetical patient described in a vignette with the person who is reading the vignette. This approach might promote the realism of the vignette and allow study participants to more closely identify with the content of the vignette.

### Patient point-of-view

The majority of vignettes contained in the included studies were targeted to participants who did not have the disease of interest. The vignettes were rarely given to participants who had the disease (seen in only three articles [21, 28, 33]). Most vignettes were written in the third-person, with hypothetical patients being called by proper names (e.g., Donna, David) or generic terms (e.g., “Mr./Mrs. X”, “he”/“she”, “the patient”). Study participants may regard vignettes written in the third-person as being more objective than first- (e.g., “I”) or second-person (e.g., “you”) vignettes. First- or second-person vignettes might evoke doubt or disbelief on the part of study participants, who would be imagining themselves as patients in what are known to be fictitious situations [4, 15]. Vignettes should be written in the third-person point-of-view to portray realistic scenarios for the reader.

### Vignette content

The characteristics of hypothetical patients varied substantially across vignettes. Even the 20 most frequently recurring characteristics across all 58 vignettes did not appear in every vignette (for example, Karlsson and colleagues used a vignette with none of the 20 characteristics [43]), while as many as nine of these characteristics appeared in some vignettes (i.e., Holroyd et al.’s vignette contained nine of the 20 characteristics among all described symptoms [41]). Two reasons account for this variation. First, some characteristics might not be relevant for the purpose of the research. For example, Hebert and colleagues investigated caregivers’ experiences with driving and dementia patients [39]. Understandably, the study’s vignette focused on symptoms related to this objective (e.g., disorientation to location), as opposed to symptoms that may not provide the reader with relevant information (e.g., paranoia). Many vignettes were drafted to meet narrow and specific study objectives. These vignettes were not developed to describe broad constellations of symptoms. Second, no guidelines exist to govern the design of clinical vignettes for AD. For example, vignettes do not have a set length. Two [25, 38] of the included studies reported their vignette length, effectively demonstrating the variability amongst the total vignette population. Cairns et al. [25] reported vignette length as ranging from 330 to 1018 words (mean 782 words), whereas Harden and colleagues [38] reported vignette length as ranging from only 60 to 80 words. Differences in length affect the amount of content in the vignettes. Longer vignettes can describe more symptoms, or they can expand on the descriptions of a select number of symptoms. However, longer vignettes will increase the amount of time required of participants to complete the study’s tasks. Increased time commitments could create disincentives to participate in research, or lead to greater amounts of missing data because participants do not complete lengthy surveys. Researchers must balance the amount of vignette content with the practical requirements of recruiting participants and obtaining complete data.

Notwithstanding vignettes developed for very narrow and specific purposes, the goal of researchers in AD or other dementias should be to promote the standardization of the content of vignettes. Standardization enhances inter-study comparability and lends itself to the production of evidence syntheses such as systematic reviews and meta analyses, which are important tools to inform clinical and policy decisions in health care [74, 75]. Many research topics in AD or other dementias lend themselves to the use of standardized vignettes. Examples include the use of proxy respondents to obtain measures of the QoL of persons with AD or to elicit attitudes to AD (e.g., should the healthcare system devote more resources to AD versus coronary heart disease?). Proxy respondents might include members of the general population, caregivers in AD, or health professionals. Careful selection of hypothetical patient characteristics is required because the content of the vignettes will shape proxy respondents’ responses to study questionnaires.

Standardized vignettes could be developed for individual research areas. For QoL, as an example, researchers could create sets of vignettes describing hypothetical patients with the characteristics that map onto the domains contained in QoL instruments such as the EQ-5D [9], SF-36 [76], and QoL-AD [68]. Any QoL research involving a certain instrument would utilize the set of vignettes developed to match the content of the instrument in question. Such purposeful selection of content helps to overcome the variability in vignettes that is motivated by the heterogeneous clinical manifestations of AD or other dementias.

Researchers should employ focus groups or one-on-one interviews to assess the content validity of newly developed vignettes. Participants for these validity studies should be drawn from the same sample frame as the persons who would be approached to participate in a ‘full’ study. These participants would read the vignettes under consideration and respond to semi-structured interview questions about content (e.g., should items be added or deleted from the vignettes), length, wording, and formatting (e.g., paragraph form versus point form). Focus groups provide a forum for participants to validate one another’s ideas and build upon each other’s thoughts. One-on-one interviews are an ideal forum for in-depth probes about the vignettes without the distractions of the group approach (e.g., dominance by one or two group members, shyness in social situations as an obstacle to giving feedback in group settings). Researchers may wish to consider a mix of focus groups and one-on-one interviews, and they should report their methods of validating their vignettes.

Researchers should assess newly developed vignettes for response consistency and vignette equivalence before using the vignettes in their studies. Response consistency is the extent to which participants provide equivalent ratings of some aspect of their personhood (e.g., their health) when asked (1) to directly rate the aspect and (2) to rate the same aspect, but this time after reading a vignette description of the aspect [77]. Vignette equivalence is the extent to which all participants rate a vignette’s description in the same way [78]. Equivalence is often assessed by verifying whether participants’ ratings of the same description remain stable after changing an inconsequential component of the vignette text (e.g., changing from a male to a female name in a third-person vignette describing mild AD).

## Conclusions

Key aspects to consider when constructing a vignette for AD or another dementia include:

1. Vignettes should be written from the third-person perspective, although the sex of the hypothetical patients described in the vignettes could be altered to match individual study participants;
2. The patient should be presented as being equal to or greater than 65 years of age; the mean patient age described in the literature is 72 years of age; and
3. Where possible, vignettes should contain standardized descriptions of hypothetical patients.

## Additional files

Additional file 1:
**Sample search string and list of similar disorders.**
Additional file 2: Tables S1-S3. Study and vignette characteristic tables. The three tables, separated by vignette focus (i.e., AD, non-AD dementia, similar conditions), summarize study objective, symptoms and behaviours described by the vignettes, point of view, and patient age and sex.
