# Supplementary material for: Identifying the components of clinical vignettes describing Alzheimer’s disease or other dementias: a scoping review
Source: BMC Med Inform Decis Mak. 2015 Jul 15;15:54. doi: 10.1186/s12911-015-0179-x (PMC4502543; doi:10.1186/s12911-015-0179-x)
Supplement: Additional file 1: — Sample search string and list of similar disorders. [file 12911_2015_179_MOESM1_ESM.docx]

**Additional file 1 – Sample search string and list of similar disorders**

Search string used to identify articles for the review in MEDLINE (inception to June 2014).

1. exp Dementia/

2. dementia.mp.

3. exp Alzheimer’s Disease/

4. alzheimer$.mp.

5. exp Vascular dementia/

6. multi-infarct dementia.mp.

7. binswanger disease.mp.

8. congophilic angiopathy.mp.

9. cerebral amyloid angiopathy.mp.

10. (cerebral autosomal dominant arteriopathy with subcortical infarcts and leukoencephalopathy).mp.

11. neoplastic angioendotheliosis.mp.

12. granular cortical atrophy.mp.

13. lewy body disease.mp.

14. frontotemporal dementia.mp.

15. pick disease.mp.

16. primary progressive aphasia.mp.

17. posterior cortical dementia.mp.

18. posterior cortical atrophy.mp.

19. progressive apraxia.mp.

20. exp Multiple Sclerosis/

21. multiple sclerosis.mp.

22. exp Parkinsonism/

23. parkinson$.mp.

24. parkinsonian disorder.mp.

25. normal pressure hydrocephalus.mp.

26. exp Progressive Supranuclear Palsy/

27. progressive supranuclear palsy.mp.

28. huntington$.mp.

29. corticobasal ganglionic degeneration.mp.

30. corticobasal degeneration/

31. mesolimbocortical dementia.mp.

32. progressive subcortical dementia.mp.

33. pantothenate kinase-associated neurodegeneration.mp.

34. hallervorden-spatz disease.mp.

35. creutzfeldt-jakob disease.mp.

36. tauopathy.mp.

37. exp AIDS Dementia Complex/

38. exp Aphasia/

39. exp Creutzfeldt Jakob Syndrome/

40. exp Neurofibrillary Tangles/

41. frontotemporal lobar degeneration.mp.

42. exp semantic dementia/

43. exp Huntingtons Disease/

44. exp Kluver Bucy Syndrome/

45. exp Dementia with Lewy Bodies/

46. exp Picks Disease/

47. 1 or 2 or 3 or 4 or 5 or 6 or 7 or 8 or 9 or 10 or 11 or 12 or 13 or 14 or 15 or 16 or 17 or 18 or 19 or 20 or 21 or 22 or 23 or 24 or 25 or 26 or 27 or 28 or 29 or 30 or 31 or 32 or 33 or 34 or 35 or 36 or 37 or 38 or 39 or 40 or 41 or 42 or 43 or 44 or 45 or 46

48. vignette.mp.

49. limit 48 to English language

50. 47 and 48 and 49

List of similar disorders

multi-infarct dementia, binswanger disease (subacute arteriosclerotic encephalopathy), congophilic (amyloid) angiopathy, cerebral autosomal dominant arteriopathy with subcortical infarcts and leukoencephalopathy, neoplastic angio-endotheliosis, subacute diencephalic angio-encephalopathy, cortical micro-infarction (granular cortical atrophy), Lewy body disease, frontotemporal dementia, pick disease, primary progressive aphasia, posterior cortical (atrophy) dementia, progressive apraxia, multiple sclerosis, Parkinson’s disease, normal pressure hydrocephalus, progressive supranuclear palsy, Huntington’s disease, corticobasal ganglionic degeneration, mesolimbocortical dementia, progressive subcortical gliosis, Hallervorden-Spatz disease, and Creutzfeldt-Jakob disease.
