# Supplementary material for: Identifying the components of clinical vignettes describing Alzheimer’s disease or other dementias: a scoping review
Source: BMC Med Inform Decis Mak. 2015 Jul 15;15:54. doi: 10.1186/s12911-015-0179-x (PMC4502543; doi:10.1186/s12911-015-0179-x)
Supplement: Additional file 2: Tables S1-S3. — Study and vignette characteristic tables. The three tables, separated by vignette focus (i.e., AD, non-AD dementia, similar conditions), summarize study objective, symptoms and behaviours described by the vignettes, point of view, and patient age and sex. [file 12911_2015_179_MOESM2_ESM.docx]

**Additional file 2 – Tables S1-S3**

Study and vignette characteristic tables. The three tables, separated by vignette focus (i.e., AD, non-AD dementia, similar conditions), summarize study objective, symptoms and behaviours described by the vignettes, point of view, and patient age and sex.

**Table S1. Study and vignette characteristics for AD**

| **Study (Author, year, country)** | **Study Objective (i.e. reason for vignette methodology)** | **Symptoms/ Behaviours Described by the Vignette(s)** | **Point of View, Age & Sex of Patient (if applicable) and Notes on Vignette** |
| --- | --- | --- | --- |
| Ayalon, 2010, Israel [21] | Evaluate attitudes toward elder mistreatment | Deterioration in memory and thinking; Delusions/false beliefs; Wandering | - 3^rd^ person  - Female/“older” patient |
| Blay, 2008, Brazil [22] | Evaluate the lay public’s (1) attitudes toward help-seeking behaviour for AD, and (2) preferences for treatment of AD | Forgetfulness (forgetting events immediately after they occur); Difficulty recognizing & identifying names of common things; Difficulty in carrying out daily tasks unassisted | - 3^rd^ person  - Male or female (70 yo)  - Symptoms described in accordance with DSM-IV and ICD 10 criteria |
| Bourkel, 2012, Luxembourg [23] | Investigate (1) how laypeople and professionals judge the rights of someone with AD, and (2) whether social distance exists toward people with AD | Extreme forgetfulness; problems learning new content, but older memories are not effected much; Difficulty concentrating; Difficulty with organizational activities & activities of daily living; Irritable; Anxious; Depressive; Faltering language skills | - 3^rd^ person  - Male or female (75 yo)  - Symptoms chosen were to describe a patient in an early stage of Alzheimer’s Dementia |
| Cairns, 2011, UK [25] | Assess the level of agreement between evaluations of ‘deprivation of liberty’ made by lawyers and those of different groups of mental health professionals | “Vignette 2” –Advanced dementia:  Agitated/ threatening behaviour; Lacking capacity to make treatment or living decisions; Depressive  “Vignette 3” – Brain injury:  Short- & long-term memory impairment; Disorientation; Paranoia; Lacking in capacity to make decisions about treatment; Denial of condition  “Vignette 9” Advanced dementia of mixed vascular and Alzheimer’s types:  Unable to understand or answer questions; Agitated behaviour; Tearfulness; Resistance to personal care; Lacking in capacity to make decisions about care and accommodation; Wandering; Responds to reassurance & distraction techniques; Depressive | - 3^rd^ person  - Male/91,80,40 yo in vignettes 2,3,9, respectively  - Information included in vignettes collected from medical notes of patients & patient interviews  - Vignette length ranged from 330 to 1018 words (average 782)  - 12 total vignettes; 3 provided in publication |
| Colenda, 1996, USA [29] | Understand treatment recommendations of physicians treating agitated dementia patients, and the variables influencing them | Physical agitation, especially concerning activities of daily living (patient specified as having never been agitated before) | - 3^rd^ person |
| Day, 1995, USA [30] | Identify the circumstances under which nurses refuse artificial nutrition or hydration for patients | Almost bedridden; Incontinent; Unable to speak comprehensibly; Refuses to eat; Unresponsive | - 3^rd^ person  - Female/75 yo |
| DeLetter, 1995, USA [31] | Develop and test a Cognitively Impaired Life Quality (CILQ) scale, to be used to quantify differences in quality of life of cognitively impaired older adults, as perceived by nursing caregivers | Unable to walk; Requires tube feeding; Incontinent; Unable to recognize family or staff | - 3^rd^ person  - Male/86 yo |
| Fazel, 2000, UK [33] | Develop a patient-centred method to assess the competence of patients with cognitive impairment to complete an advance directive | “Vignette 2”:  Forgetful and occasionally confused; Deteriorating memory; Recognizes relatives and nursing home staff  “Vignette 3”:  Memory problems (recent and past events); Difficulty with activities of daily living | - 2^nd^ person  - 3 vignettes; all were read to each respondent (two were used to describe Alzheimer’s dementia and are included in the current review)  - Vignettes included treatment options |
| Fortinsky, 1997, USA [36] | Examine how physicians diagnose cognitive dysfunction (i.e. methods used in diagnosis of dementia) | Mild:  Starting to forget names and telephone numbers; Got lost on a familiar route; Withdrawal from some routine social activities  Moderate:  Forgetting many names and telephone numbers; Difficulty participating in conversation; Needs assistance with activities of daily living; Increasingly agitated | - 3^rd^ person  - Female/72 yo  - Two vignettes designed; one for “mild symptoms” and one for “moderate symptoms” |
| Hebert, 2003, USA [39] | Investigate caregivers’ experiences with dementia patients and driving | Slowed thinking and poor short-term memory; Often misplaces items; Slow to react when driving; Seems nervous when driving; Has gotten lost on familiar routes | - 3^rd^ person  - Male/80 yo |
| Holroyd, 1996, USA [41] | Examine the attitudes of older adults toward being told the diagnosis of AD | Trouble with memory (including recalling names); Confused about the date; Difficulty with activities of daily living; Has noticed change in condition and is upset because of it; Memory will deteriorate over time and ability to think will also worsen; Will be unable to carry out activities of daily living; May experience wandering; May become increasingly irritable and moody; May not recognize family members; May become unable to speak; May experience delusional behaviour | - 3^rd^ person  - Female/66 yo  - Vignette begins by describing current condition of patient, and also describes the likely future outcomes  - 2 vignettes (one for AD, one for cancer; AD vignette included in the current review) |
| Kalaitzaki, 2012, Greece [42] | Examine disease identification, emotional reactions, and cognitive perceptions of AD | Cannot recognize many friends/family members; Increasingly sensitive in emotional reactions; Difficulty with activities of daily living; Trouble speaking; unable to think of appropriate words; Repeats self often | - 3^rd^ person  - Female/67 yo  - Symptoms describe moderate AD in accordance with DSM-IV criteria |
| Karlsson, 2000, Sweden [43] | Explore the reasons for nurses to use physical restraints with elderly patients | Incontinent; Able to communicate with caregivers, relatives, etc.; Reduced ability to walk; has fallen several times | - 3^rd^ person  - Male/78 yo |
| Low, 2009, Australia [46] | Explore the recognition of dementia and beliefs about prognosis, causes, and risk reduction in the Australian general public | Mild:  Very forgetful; Confusion about daily plans; Increasing tendency to lose things; Decrease in activity; Withdrawal of some social activities; Trouble remembering names of familiar people  Moderate:  Deteriorating memory; very poor short term memory; Repeats stories; Discusses distant past as if it were recent; Occasionally confused, angry, and aggressive; Unable to complete activities of daily living; Decline in self-maintenance; Withdrawal from social activities; Delusional | - 3^rd^ person  - Male or female (75 yo)  - 2 vignettes; one describing mild AD symptoms and one describing moderate AD symptoms  - Symptoms and behaviours of patients with AD described in accordance with DSM-IV criteria |
| Maeck, 2007, Germany [47] | Examine whether primary care competency in early diagnosis of dementia changed between 1993 and 2001 | Vignette 1a/b:  Tendency to lose things; Deterioration of short-term memory; Trouble following a conversation; causes nervousness and insecurity; Steady decline in ability to concentrate  Vignette 2a:  Diffuse dizziness and headaches; Deteriorating memory; Decline in ability to concentrate; Wandering; Delirious; Often misplaces items and blames others; Periods where patient behaves as before; Disoriented to time, and to some degree, place  Vignette 2b:  Progressive decline in memory and concentration; Unable to process new information; Difficulty with problem solving; causes insecurity; Decrease in activity; Often misplaces items; Often repeats self; Difficulty finding with conversation; Disorientation to time, and to some degree, place | - 3^rd^ person, with 1^st^ person quotes from patient  - Male or female (70 yo)  - 4 vignettes total; 1a & 1b described mild cognitive impairment with suggestion of the beginning of dementia in accordance with ICD-10 criteria (differed in gender only); 2a & 2b described a female patient suffering from moderate dementia (differed in suggestion of etiology: 2a=moderate dementia of vascular origin, 2b=Alzheimer’s dementia) |
| Manthorpe, 2010, UK [48] | Explore the need for healthcare practitioners to respect the dignity of older patients | Problems with memory; Difficulty managing finances/accounts; Inappropriate behaviour | - 3^rd^ person  - Male  - Participants were presented with 5 different vignettes (dementia diagnosis, lying, sex and intimacy, incontinence, end of life care) (dementia diagnosis vignette, which suggested AD as the etiology, is included in the current review) |
| Ploeg, 2009, Canada [50] | Examine where older adults seek help with caring for a parent with dementia, and the factors associated with identification of community, health, and support services | Vignette 1:  Patient is taking more pills than they should  Vignette 2:  Unable to complete activities of daily living  Vignette 3:  Very confused; Cannot be left unsupervised/ unattended to | - Patient described in 3^rd^ person, vignettes read in 2^nd^ person to respondent  - Vignette 1: female, vignette 2: male, vignette 3: female |
| Richter, 2001, Germany [51] | Investigate how end-of-life treatment decisions are influenced by sociopolitical and cultural circumstances | Cannot answer simple questions coherently; Understands only simple commands; Occasionally agitated; Cannot perform activities of daily living; Wandering ; Difficulty remembering names; Occasionally does not recognize family members; Incontinent | - 3^rd^ person  - Male/82 yo |
| Sbordone, 1986, USA [52] | Determine whether psychologists can recognize underlying neurological disorders in patients who present with disturbances in psychological functioning | Very nervous; Increasing difficulty with performance at work; Deterioration of short-term memory; Denial of problems with cognition | - 3^rd^ person  - Male/58 yo  - Four vignettes (on different conditions) presented to each respondent (vignette describing AD included in the current review) |
| Wadley, 2001, USA [56] | Explore the influence of diagnostic labeling on attributions, emotions, and helping intentions toward patients | Inappropriate/ rude behaviour; Disoriented to time and situation; Deteriorating memory | - 3^rd^ person; vignette was to be read as if patient was the respondent’s parent  - Male or female  - 1 of 9 variants of the vignette was published and included in the current review (incongruent behaviour and AD label) |
| Werner, 2004, Israel [57] | Explore laypersons’ beliefs about the helpfulness of interventions for AD | Vignette 1:  Unable to recognize familiar people; Deteriorating memory; Often misplaces items; Often forgets tasks; Difficulty with new routes  Vignette 2:  Unable to recognize familiar people, including family sometimes; Deteriorating memory; Trouble with speech; Agitated, suspicious | - 3^rd^ person  - Male/71 yo  - Two vignettes; varied by the stage of AD (vignette 2 describes advanced AD)  - Symptoms described AD in accordance with DSM-IV criteria |
| Werner, 2008, Israel [58] | Examine whether lay peoples’ stigmatic beliefs toward people with AD vary by the type of residence in which the person lives | Difficulty recognizing family; Difficulty with communication; often responds inappropriately to questions; Sometimes agitated/restless; Often repeats self; Forgets familiar routes; Pacing | - 3^rd^ person  - Male/78 yo  - Two versions of the vignette administered; varied by place of residence of patient (community vs. nursing home)  - Symptoms described AD in accordance with DSM-IV criteria |
| Werner, 2004, Israel [59] | Examine laypersons’ emotional reactions to a person with dementia | Agitated and aggressive; Suspicious; Denial of condition; Disoriented to time and place | - 3^rd^ person  - Male/75 yo  - Symptoms described AD in accordance with DSM-IV criteria |
| Xie, 2012, Canada [13] | Investigate whether the general public is able to differentiate between stages of AD, and whether the general public’s proxy HRQoL estimates correlate with their own health status | Mild:  Forget recent events; Forget personal details; Decline in ability to concentrate; Difficulty with organizational activities; Oriented to time and place  Moderate:  Unable to remember some important facts, personal details, and names of relatives; Sometimes will forget time and place; Might need assistance with maintenance of self  Severe:  Unable to perform activities of daily living; Experience periods of agitated behaviour; Delusional | - 2^nd^ person  - 3 vignettes; mild, moderate, severe AD |

AD=Alzheimer’s disease, yo=years old, N/A=Not applicable HRQoL=Health-Related Quality of Life, DSM= DSM=Diagnostic and Statistical Manual of Mental Disorders

**Table S2. Study and vignette characteristics for Non-AD Dementia**

| **Study (Author, year, country)** | **Study Objective (i.e. reason for vignette methodology)** | **Symptoms/ Behaviours Described by the Vignette(s)** | **Point of View, Age & Sex of Patient (if applicable) and Notes on Vignette** |
| --- | --- | --- | --- |
| Burgio 1995, USA [24] | Assess geriatric nurses’ acceptance of different treatments for behavioural disturbances in older adults | Cognitive capacity:  Patient depicted as either occasionally forgetful but organized and lucid (i.e. without dementia) or as suffering from dementia; Patient with dementia depicted as having “good and bad days”, and being forgetful/disoriented and unable to recognize caregivers on bad days  Living/Caregiver:  Patient described as living in the community with family caregivers or in a nursing home  Presenting problem:  Patient displayed either physical aggression, verbal abuse, or noncompliance | - 3^rd^ person  - Female/75 yo  - 12 vignettes generated; cognitive capacity, living situation/ caregiver, and presenting problem were varied  (vignettes not published, but contents were described) |
| Cheng, 2011, China [26] | Explore whether brief educational scenarios (i.e. vignettes) could reduce stigma, and also whether the inclusion of a diagnostic label (i.e. defining the condition as dementia) further reduces that stigma | Vignette 1:  Deterioration of memory; Tendency to repeat self while talking; Forgetfulness of facts  Vignette 2:  Poor memory; Tendency to get lost; Irritable; tendency to lose temper over minor issues; Suspicious; thinks people are stealing possessions; causes patient to be unhappy most of the time | - 3^rd^ person  - Vignette 1: Male/80 yo  - Vignette 2: Female |
| Clare, 2012, UK [28] | Evaluate the utility of vignettes as a tool to assess the ability of a patient with dementia to identify and correctly attribute symptoms of dementia and suggest appropriate ways of managing them | Established dementia:  Forgetfulness; Repeats self without realizing; Deterioration of memory; Disoriented to time and situation  Early-stage dementia:  Difficulty in organizational/daily tasks; Difficulty with speech (i.e. forgetfulness of words); Conscious worry about memory problems | - 3^rd^ person  - Male or female  - 3 vignettes used; one for healthy aging (90 yo), one for early-stage dementia (72 yo), one for established dementia (65 yo) (dementia vignettes included in the current review)  - Each vignette prepared as two versions, differing only in patient’s gender |
| Hamilton-West, 2010, UK [37] | Develop a scale that can assess laypersons’ perceptions of dementia and determine whether representations of illness assessed via this scale are related to intentions to seek help on behalf of a relative with potential signs of dementia | Mild:  Sometimes confused and short-tempered; Confusion with words sometimes; Using incorrect household items for certain tasks; Repetition of self; Withdrawal of social activities  Moderate:  Confusion with words; Short-tempered and easily agitated; Deteriorating memory; Decline in self-maintenance and completion of activities of daily living; Not sleeping at night | - Vignette written in 1^st^ person, describing a female patient in 3^rd^ person (i.e., meant to be read as respondent talking about his/her mother)  - 2 vignettes; one for mild and one for moderate condition  - Vignettes matched for word count |
| Herron, 2013, UK [40] | Explore support workers’ knowledge and skill in the area of mental health issues in older persons with intellectual disabilities | Cannot speak – communicates well with gestures and facial expressions; Irritable behaviour; Withdrawal from previously enjoyed activities; Incontinence; Confusion; Loss of skills; Memory impairment | - 3^rd^ person  - Male, 72 yo  - Three vignettes which described progression of dementia indicators (description of what each of the vignettes included was published and is included here) |
| La Fontaine, 2007, UK [45] | Explore perceptions of aging, dementia, and age-associated mental health issues amongst Punjabi persons in Britain | Mild dementia:  Change in mood – increasingly sensitive to emotions; Does not care as much about personal appearance as before; Withdrawal from social activities; Some difficulty remembering names; Difficulty with recall of recent events  Severe dementia:  Withdrawn and inactive; Does not care about appearance; Very forgetful – often unable to recognize family members; Poor short term memory; Irritable; Talking to self; Difficulty with activities of daily living; Mistakes times of day | - Written in 1^st^ person, describing patient in 3^rd^ person  - Female  - 3 vignettes; normal aging, mild dementia, severe dementia (dementia vignettes included in the current review) |
| Normann, 1999, Norway [49] | Explore the attitudes of registered nurses in a northern Norwegian country toward patients with severe dementia | Disoriented to time, place, and situation; Frequently repeats restless movements; Wandering; Difficulty communicating/ difficulty with speech; Paranoia; Confusion | - 3^rd^ person  - Female/84 yo |
| Trickey, 2000, UK [53] | Investigate attitudes and knowledge of primary care nurses toward assessing and managing patients with symptoms of dementia | Increasingly forgetful – forgets names of familiar objects; Increasingly agitated; Difficulty with some activities of daily living; Withdrawal from social activity | - 3^rd^ person  - Female/86 yo |
| Uppal, 2014, UK [54] | Investigate perceptions and understanding of dementia in Sikh persons in the UK | Withdrawal from social activity; Poor short term memory; Disoriented to time; Difficulty communicating with others and mumbles to self | - 3^rd^ person  - Female |
| Werner, 2004, Israel [60] | Examine characteristics of physician-patient-caregiver encounters where dementia is present | Difficulty with activities of daily living; Inactive; Delusional; Difficulty remembering names | - Patient described in 3^rd^ person; vignette presented to respondent in 2^nd^ person as if they were giving the patient an examination  - Female/76 yo  - Two vignette versions; one in which patient is calm during the examination, and one in which patient is described as “agitated and uncooperative” during the examination |
| Wijeratne, 2009, Australia [61] | Determine recognition of mental disorders in older people, intentions regarding investigation, referral, treatment, and beliefs about prognosis by general practitioners | Poor short-term memory; Asks same questions repeatedly; Often misplaces items; Difficulty keeping track of a conversation; Lost on familiar routes; Denial of condition; Ill-tempered | - 3^rd^ person  - Male/75 yo  - 4 vignettes included in package; one each for coronary heart disease, depression, delirium, and dementia (dementia vignette included in the current review) |

AD=Alzheimer’s disease, yo=years old, ICD=International Classification of Diseases

**Table S3. Study and vignette characteristics for Other Conditions**

| **Study (Author, year, country)** | **Study Objective (i.e. reason for vignette methodology)** | **Symptoms/ Behaviours Described by the Vignette(s)** | **Point of View, Age & Sex of Patient (if applicable) and Notes on Vignette** |
| --- | --- | --- | --- |
| Ciliberto, 1981, USA [27] | Explore the hypothesis that nurses presented with the same patient characteristics (i.e. mental confusion & anxiety) in a younger and older patient are more likely to (1) diagnose (with organic brain syndrome), (2) recommend institutionalization for, and (3) give a negative prognosis for, older patients | Insomnia; Wandering; Error in judgment (i.e. placing household items in incorrect places); Disregard for personal maintenance/cleanliness; Decline in short- and long-term memory (“especially auditory”); Depression; Anxiety; Increasing irritability; Loses interest in activities which require active participation | - 3^rd^ person  - Male/68 or 28 yr  - 2 vignettes used; exact same vignette except for age of patient |
| Ekman, 2007, Sweden [32] | Collect primary data on health utilities in different stages of mild cognitive impairment and dementia | Consistent problems with slight forgetfulness; Unable to recall recent events completely; Difficulty recalling names and words; Difficulty placing past events in a chronological context; Ability to carry out activities of daily living, although slightly impaired because of memory problems | - 2^nd^ person  - 4 vignettes describing health states with cognitive impairment typical in different stages of dementia (based on Clinical Dementia Rating scale) were used; only one was published and included in the current review (mild cognitive impairment) |
| Fick, 2007, USA [34] | Assess nurses’ identification of delirium superimposed on dementia | Increasing memory problems over the last year; Has gotten lost many times on familiar routes; Difficulty completing activities of daily living; Does not notice people speaking to her; Constantly tired and falls asleep at inappropriate times; Disoriented to time and place; Does not recognize nurse; Will not eat or take medication | - 3^rd^ person  - Female, 74 yr  - 5 different vignettes describing different subtypes of delirium superimposed on dementia (1 vignette published and included in current review) |
| Fick, 2013, USA [35] | Describe nursing home staff members’ knowledge of delirium detection and the most common delirium causes. | Increasing memory problems over past year; Has gotten lost many times on a familiar route; Difficulty recalling names; Difficulty completing activities of daily living; Confusion; Paranoid behaviour – violent; Disoriented to time and place | - 3^rd^ person  - Female, 83 yr  - 5 different vignettes describing dementia, delirium superimposed on dementia, and delirium (1 vignette published and included in current review) |
| Harden, 2004, USA [38] | Determine characteristics of epilepsy which produce avoidant behaviour in the workplace | Walks slowly, with assistance of a cane; Difficulty with hand movements; Speaks slowly; Needs assistance with some activities of daily living | - 3^rd^ person (described as reader’s coworker)  - Female/49 yr  - 3 vignettes; described depression, MS, epilepsy (MS vignette included in the current review)  - Vignettes were 60 to 80 words in length |
| Korner-Bitensky, 2011, Canada [44] | Assess variations in occupational therapists’ management of post-stroke cognitive impairment | Alert and oriented to people and time, but not place; Easily distracted; Difficulty expressing ideas; has to search for words | - 3^rd^ person  - Multiple vignettes  - Vignettes not published; descriptions/statements about the vignettes included |
| VonDras, 2009, USA [55] | Explore how age and HRQoL might be related to the identification and appraisal of cognitive impairment symptoms and associated prevention beliefs | Easily confused; Gets lost on familiar routes; Deteriorating short-term memory; Cannot recall names | - 3^rd^ person  - Male or female (70 yr) |

N/A=Not applicable, yr=years, HRQoL= Health-Related Quality of Life, MS=Multiple sclerosis
